# Salivary Fluoride Levels after Use of High-Fluoride Dentifrice

**DOI:** 10.1155/2015/302717

**Published:** 2015-03-03

**Authors:** Glauber Campos Vale, Priscila Figueiredo Cruz, Ana Clarissa Cavalcante Elvas Bohn, Marcoeli Silva de Moura

**Affiliations:** Federal University of Piauí, 6404-550 Teresina, PI, Brazil

## Abstract

The aim of the study was to evaluate salivary fluoride (F) availability after toothbrushing with a high-F dentifrice. Twelve adult volunteers took part in this crossover and blind study. F concentration in saliva was determined after brushing with a high-F dentifrice (5000 *µ*g F/g) or with a conventional F concentration dentifrice (1100 *µ*g F/g) followed by a 15 mL distilled water rinse. Samples of nonstimulated saliva were collected on the following times: before (baseline), and immediately after spit (time = 0) and after 1, 2, 3, 4, 5, 10, 15, 20, 30, 45, 60, 90, and 120 min. F analysis was performed with a fluoride-sensitive electrode and the area under curve of F salivary concentration × time (*µ*g F/mL × min^−1^) was calculated. At baseline, no significant difference was found among dentifrices (*P* > 0.05). After brushing, both dentifrices caused an elevated fluoride level in saliva; however salivary F concentration was significantly higher at all times, when high-F dentifrice was used (*P* < 0.01). Even after 120 min, salivary F concentration was still higher than the baseline values for both dentifrices (*P* < 0.001). High-F dentifrice enhanced the bioavailability of salivary F, being an option for caries management in patients with high caries risk.

## 1. Introduction

The use of fluoridated toothpaste is considered the main reason for the reduction in caries prevalence that was observed in the last decades [1]. This method of caries prevention associates the disorganization or removal of biofilm by mechanical act of brushing with the uptake of fluoride (F) to the oral environment [2]. In fact, elevated levels of fluoride have been found in saliva, plaque, and the oral soft tissues after use of fluoridated toothpaste, which persist at potentially active concentrations for hours [3].

F bioavailability in saliva has been used to estimate the anticariogenic potential of fluoridated toothpaste. This is because, from the saliva, F diffuses into the biofilm and its fluid, which acts in the deremineralization processes on tooth-biofilm interface [4, 5]. However, the intraoral F retention or substantivity is mainly influenced by user-related factors, which consists in biological aspects, such as salivary flow and salivary clearance, and behavioural aspects, such as frequency and duration of brushing, amount of toothpaste used, and postbrushing rinsing behavior [6].

The use of toothpaste with high-F concentration (5000 ppm F) appears to be effective in controlling root caries in front of a conventional dentifrice (1100 ppm F) in elderly patients [7]. This is relevant, because there seems to be an increased risk of root lesions in the elderly, since the life expectancy is increasing over the years and a greater number of teeth and exposed root surfaces are observed in these individuals mouths [8]. Likewise, other group age patients with high caries risks can also benefit from 5000 ppm F dentifrice. Nordström and Birkhed [9] showed that caries-active adolescents using the 5,000 ppm F toothpaste had significantly lower progression of caries compared to subjects using the 1,450 ppm toothpaste after 2 years.

However, little is known about the salivary bioavailability of F after use of a high-fluoride dentifrice in order to have more accurate information on the recommendation of toothpaste with high-fluoride concentration for patients with high caries risk.

## 2. Methods

### 2.1. Experimental Design

In this crossover and blind study, approved by the local Research and Ethics Committee (Protocol 483.913), twelve adults brushed their teeth for 1 min with either a high-F concentration dentifrice (5,000 *μ*g F/g) or a conventional F dentifrice (1,100 *μ*g F/g), both as NaF, followed by a water rinse. Nonstimulated saliva samples were collected immediately before brushing and up to 120 min after brushing. Salivary F concentration was determined using F electrode. The response variables considered were the salivary F concentration at each time, the maximum salivary concentration (*C*_max⁡_), and the area under the curve (AUC) of salivary F concentration × time (*μ*g F/mL × min^−1^). For statistical comparisons, each volunteer was considered as an experimental unit.

### 2.2. Volunteers

Twelve healthy adult volunteers (mean age 21.3 years) signed a written informed consent prior to their participation in the study. They had good general and oral health, normal salivary flow rate, and complete natural dentition.

### 2.3. Dentifrices

The dentifrices used were Pharmacia-manipulated containing 5,000 *μ*g F/g or 1,100 *μ*g F/g, both as NaF, in a silica base and pepper-mint flavour.

### 2.4. Experimental Protocol

Volunteers were randomly assigned using a computer-generated list to start the experiment using one of the dentifrices. According to the crossover design, at the end of the experiment all volunteers had used both dentifrice concentrations. Volunteers were instructed to avoid F-rich foods and beverages during the experiment, but no recommendation was made with respect to brushing habits and no F-free washout period was allowed. All saliva collection started at least 2 hrs after breakfast, and during the 2 hr collection, volunteers refrained from speaking, eating, or drinking [10]. Volunteers were asked to brush for 1 min with 1.0 g of the assigned dentifrice after expectorating the foam, and they rinsed their mouth with 15 mL of distilled water for 10 sec. Unstimulated saliva samples were collected before (baseline) and immediately after brushing/rinsing (time zero) and at times 1, 2, 3, 4, 5, 10, 15, 20, 30, 45, 60, 90, and 120 min after brushing. The amount of saliva collected was at least 0.25 mL, sufficient for analysis by the technique used.

### 2.5. Determination of F Concentration in Saliva Samples

Saliva samples were clarified by centrifugation for 2 min at 3,024 ×g and diluted with TISAB II (1 part of saliva : 1 part of TISAB II). The fluoride concentration in the samples was calculated from the linear regression of the calibration curves obtained with standard fluoride concentrations ranging from 0.125 to 32 ppm F. The accuracy of the readings was assessed by testing solutions with known concentrations of fluoride. The fluoride present in the standards and the samples was determined by F ion specific electrode (Orion 96-09, Research Inc.) coupled to a pH/fluoride analyzer equipment. The area under the curve (AUC) of salivary F concentration × time (*μ*g F/mL × min^−1^) was calculated from time zero to 120 min using the program Origin 8.0 (Microcal Software, Inc., Northampton, MA, USA).

### 2.6. Statistical Analysis

The effect of dentifrices was tested using a post-ANOVA Tukey test, considering volunteers as a source of variation. In order to fit the assumptions of normal distribution of errors and equality of variances, data were log transformed. All analyses were performed using the SAS software (SAS Institute Inc., version 9.0, Cary, NC, USA), with *P* level fixed at 5%.

## 3. Results

**Figure 1:**
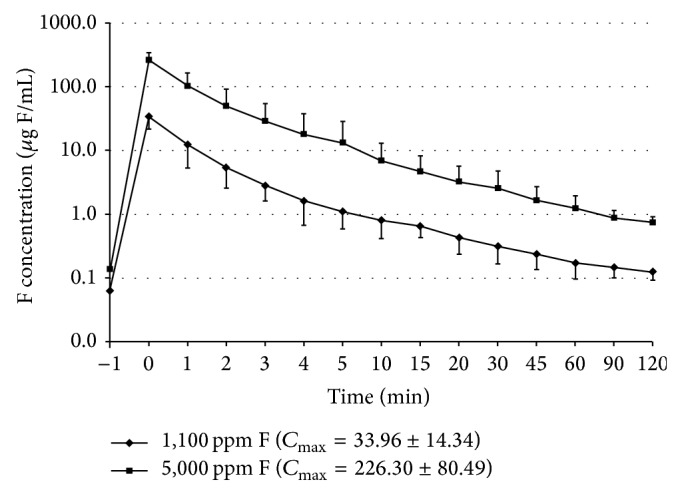
Salivary F concentration (mean ± SE) after the use of 1,100 or 5,000 *μ*g F/g dentifrices. Baseline values are plotted at time −1. F concentration values are plotted in log scale. Values were statistically different between dentifrices except in baseline (*P* < 0.001). *C*_max⁡_ (mean ± SE) is shown at the legend.

Figure 1 shows the curves of salivary F concentration × time for both dentifrices. At baseline no significant difference was found among groups (*P* > 0.05). Immediately after brushing, salivary F concentrations significantly increased for both dentifrices; however salivary F concentration was significantly higher at all times, when high-F dentifrice was used (*P* < 0.001).

Regardless of the dentifrice used, the maximum concentration (*C*_max⁡_, Figure 1) was found immediately after use of the product (time = 0) and even after 120 min, salivary F concentration, ranging from 0.12 to 0.61 *μ*g F/mL, was still higher than the baseline values for both dentifrices (*P* < 0.001).

**Figure 2:**
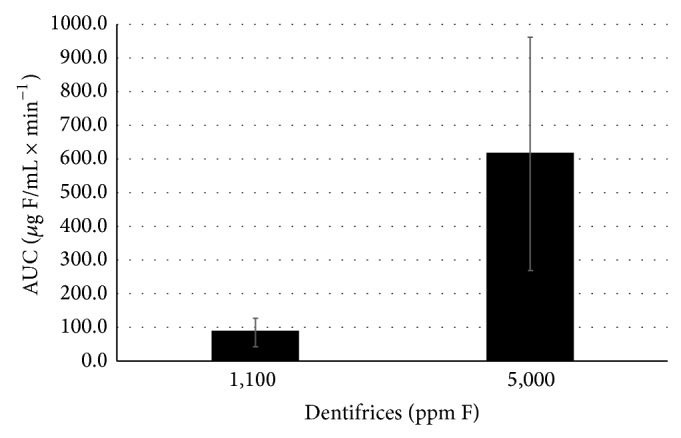
Mean ± SE of the area under curve (AUC) of salivary F concentration × time (*μ*g F/mL × min^−1^) according to the groups tested. Groups were statistically different (*P* < 0.001).

The AUC of salivary F concentration × time is shown in Figure 2. The highest F availability was observed by the use of the 5,000 *μ*g F/g dentifrice in comparison with conventional one (*P* < 0.001).

## 4. Discussion

Salivary F concentration is considered an indicator of F availability in fluid phase around tooth; thereby the evaluation of this parameter is relevant, especially regarding new products or prevention therapies based on different F concentrations.

The fast initial decrease in salivary F (Figure 1) in both dentifrices was already reported by Duckworth and Morgan [11], who reported that, after brushing with a fluoride dentifrice, salivary fluoride decreased in two distinct phases: a first initial phase which lasted for 40–80 min and a second slow phase lasting for several hours. Equally, in the present study, the most rapid decrease in salivary fluoride concentrations occurred during the 30 min after procedure and then slow gradual drop during subsequent 120 min of experiment. However, two hours after brushing, F levels in saliva were still higher than baseline (Figure 1), in agreement with other studies [10–12], showing that a longer time is necessary for the salivary F concentration to return to the baseline values.

Although both dentifrices showed the same curve pattern (Figure 1), higher values of F in saliva were found when 5,000 ppm F fluoride dentifrice was used in all times evaluated. Likewise higher amount of F would be present in approximal fluid plaque, since a positive correlation between the F concentration in dentifrices and the F concentration in plaque was previously reported [12]. Indeed, brushing with F dentifrice increases the F levels in the whole plaque [13, 14] and in the fluid [14] even 10 h or more after brushing.

It has been proved that clearance of fluoride from the mouth is affected by salivary flow rate of an individual, swallowing frequency, and factors which aid in retention of fluoride in the mouth [15]. However, since we compared both dentifrices in the same subjects in a crossover design, all these factors were probably almost the same during test period. Furthermore, all subjects have a normal salivary flow rate. Although some studies show that postbrushing water rinsing reduces the retention of fluoride in dental plaque [16], we decided to rinse with water in our protocol, since it is the most common practice of individuals.

The higher values of F in saliva after use of 5000 ppm F dentifrice, confirmed by AUC being approximately 7x higher than 1100 ppm F dentifrice (Figure 2), are important regarding root caries, since it has been shown that more fluoride is needed for remineralization of roots than for enamel [17]. In vitro studies reported that a 10 times higher concentration of fluoride was required to achieve caries inhibition in dentine compared to in enamel [18]. Furthermore, the results of present study also corroborate the use of a dentifrice containing 5,000 ppm F for the management of enamel caries in adolescents and adults with high caries risk [9], since the use of dentifrice containing fluoride is a feasible, cost-effective, and convenient method.

Considering that the anticaries effect of dentifrice is related with F concentration, high-F dentifrice enhanced the bioavailability of salivary F, being an option for caries management in patients with high caries risk.
